# Adjuvant Therapy in Renal Cell Carcinoma—Past, Present, and Future^☆^

**DOI:** 10.1053/j.seminoncol.2013.05.004

**Published:** 2013-08

**Authors:** Tobias Janowitz, Sarah J. Welsh, Kamarul Zaki, Peter Mulders, Tim Eisen

**Affiliations:** a Department of Oncology, Cambridge University Biomedical Research Campus, Addenbrooke’s Hospital, Cambridge, United Kingdom; b Department of Urology, Nijmegen University Hospital, Nijmegen, Netherlands

## Abstract

To date, no effective adjuvant treatment for renal cell carcinoma (RCC) has been described, but research in this area is important since the 5-year relapse rate for intermediate- and high-risk early-stage RCC is 30%–40%. Metastatic RCC can be treated successfully with immune therapy and targeted therapy. Adjuvant trials with immune therapy have been conducted, but they reported no benefit in disease-free survival, and clinical trials with targeted agents have not yet reported results. Further advances in our understanding of the molecular pathogenesis of RCC will identify additional potential targets for adjuvant treatment trials. Future challenges will consequently include target identification, as well as trial design to answer multiple trial questions concurrently, comprehensively, and economically. We review the past efforts, summarize the current adjuvant clinical trial landscape, and consider the challenges in adjuvant trials for RCC. Additionally, we identify potential future adjuvant trial treatments and propose an alternative design for future adjuvant clinical trials.

Global cancer statistics demonstrate an approximate annual incidence for renal cell carcinoma (RCC) of 271,000 cases in 2008.^1^ The incidence of RCC is rising. Within the male population of developed countries it is now the sixth most common cancer, with an estimated 111,100 new cases and a mortality of 43,000 in 2008.^2^ Active and passive smoking, hypertension, and obesity have been identified as risk factors for RCC and contribute to the increasing incidence. In addition, increasingly common use of medical imaging, such as ultrasonography, magnetic resonance imaging (MRI), and computed tomography (CT), has led to rising incidental detection rates of RCC.^3^ Consequently, many patients are now diagnosed with asymptomatic early-stage RCC. A 2008 quantitative analysis of the US National Cancer Data Base revealed that 50.6% of patients had stage I, 26.7% stages II and III, and 22.7% stage IV (metastatic) RCC at presentation.^4^

Due to the increasing number of patients with stages I–III RCC, optimizing the management of early-stage RCC is one of the key priorities in the oncological clinical practice. It is well established that radical surgical resection is curative for a proportion of these patients. This surgery can be performed in a nephron-sparing procedure with optional regional lymph node dissection, or by open or laparoscopic nephrectomy. After surgery, patients with stage I RCC have a 5-year survival of>90%, but the 5-year relapse rate after surgical excision in patients with stage II or III disease is 30%–40%.^5^ The median time to relapse is 18 months and the majority of relapses occur within 3 years of surgical resection.

In addition to surgical management, relapse risk reduction through adjuvant therapy is thus a very important goal in patients with intermediate- and high-risk early-stage RCC. However, despite significant efforts, no effective adjuvant therapy has been developed to date. This is in contrast to a number of clinically proven therapies for stage IV RCC.

This publication discusses the past, present, and future of adjuvant treatment in RCC. It summarizes risk stratification prior to adjuvant trial enrollment, important completed negative adjuvant clinical trials with a focus on immune therapies, the current adjuvant clinical trial landscape with a focus on targeted anti-angiogenic therapies, and potential future trial medications and trial designs.

## RISK STRATIFICATION PRIOR TO ADJUVANT TREATMENT IN RCC

When considering adjuvant treatment, correct patient selection is very important. As described above, some patients with early-stage RCC have a relapse risk of up to 40% and could therefore benefit substantially from successful adjuvant treatment. On the other hand, unnecessary treatment exposes patients to the risks of the therapies and their unwanted side effects and this risk must be minimized. Several predictive and prognostic scoring systems have been developed that can guide the enrollment of patients into adjuvant trials in RCC.

For localized disease, the stage, size, grade, and necrosis (SSIGN) score (also known as the Leibovich score), developed at the Mayo Clinic, is particularly useful for predicting 5-year metastasis-free survival.^6,7^ It was initially derived by correlating the relapse rate of 1,801 patients over a mean period of 9.7 years to the category of the primary tumor, regional lymph node status, tumor size, and the presence or absence of tumor necrosis on histopathological examination. Tumor necrosis is not clearly defined and is not consistently included in histopathological reports at all centers and thus presents a small limitation of the SSIGN score. Based on the final SSIGN score, patients are classified into low-, intermediate-, or high-risk categories for disease relapse.

The Integrated Staging System developed at the University of California Los Angeles (UISS) defines low-, intermediate-, or high-risk prognostic groups based on tumor, node, metastasis (TNM) staging, Fuhrman grade, and Eastern Cooperative Oncology Group (ECOG) performance status. It is validated for classification of patients with localized and metastatic disease.^8,9^ In a direct comparison within a small patient cohort, the UISS system showed a slightly inferior accuracy in stratifying relapse risk of patients with clear cell RCC than the SSIGN system.^10^ Among the current adjuvant clinical trials there are examples of trials based on either system and their analyses will further guide future risk stratification.

## GENERAL CONSIDERATIONS FOR ADJUVANT CLINICAL TRIALS IN RCC

**Figure 1:**
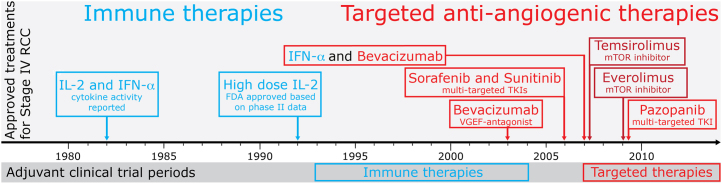
Timeline for licensed therapies for metastatic RCC and correlating adjuvant clinical trials. Immune therapies preceded targeted anti-angiogenesis therapies both in terms of approval for treatment of metastatic disease and in research for adjuvant benefit. Adjuvant trials with immune therapy were not successful and those with targeted agents are not completed yet. FDA, US Food and Drug Administration; IFN, interferon; IL-2, interleukin 2; mTOR, mammalian target of rapamycin; TKI, tyrosine kinase inhibitor; VEGF, vascular endothelial growth factor. Adapted with permission of Future Medicine Ltd.^48^

Apart from accurate risk stratification and correct inclusion and exclusion criteria for adjuvant clinical trials, the choice of treatment is paramount. In general, adjuvant treatment in oncology is guided by effective treatments for metastatic disease. Stage IV RCC has been treated successfully with immune therapy and, more recently, with targeted therapies, such as vascular endothelial growth factor (VEGF) receptor–targeted tyrosine kinase inhibitors (TKIs), VEGF antagonists, and mammalian target of rapamycin (mTOR) inhibitors. Figure 1 summarizes the timeline of licensed medications for treatment of stage IV RCC and the corresponding timeline for past and current adjuvant clinical trials. These are discussed in detail below.

### Completed Adjuvant Clinical Trials in RCC Using Immune Therapy—The Past I

Before the clinical introduction of targeted therapies, cytokine-based immunotherapy with interleukin-2 (IL-2) and/or interferon-alfa (IFN-α) was the standard therapy for stage IV RCC (Table 1). Treatment using IL-2 alone, IFN-α alone, or IL-2 in combination with IFN-α achieved objective response rates (RRs) between 15% and 31% in stage IV RCC.^11–14^ These results formed the basis for several adjuvant clinical trials with immune therapies in RCC. Theoretically, immune therapy could be considered most effective in small-volume disease. The adjuvant setting is therefore particularly relevant as it can be considered treatment of potential micro-metastatic disease.

None of these adjuvant trials, however, demonstrated statistically significant improvement of disease-free survival (DFS) or overall survival (OS) (see Table 1 for details). Two adjuvant trials using IFN-α,^15,16^ as well as one study that used high-dose bolus IL-2,^17^ were negative. The latter study was designed and powered to show an improvement in predicted 2-year DFS from 40% for the observation group to 70% for the treatment group. Early closure occurred when an interim analysis determined that the 30% improvement in 2-year DFS could not be achieved despite full accrual. Combination treatment of IL-2 and IFN-α equally failed to improve DFS in one adjuvant trial.^18^

The combination of cytokines with 5-fluorouracil (5-FU) enhances objective RRs to between 18% and 39% in metastatic RCC^19,20^; however, it failed to improve DFS in the adjuvant setting.^21,22^ In one randomized adjuvant trial, triple combination therapy using IL-2, IFN-α, and 5-FU was associated with significant toxicity (92% of patients had grade 2 and 41% had grade 3 toxicities, leading to 35% of patients not completing treatment) but resulted in no benefit in DFS or OS.^22^

All of the clinical trials used observation as the control arm and all excluded patients with metastatic disease apart from the trial on high-dose IL-2.^17^ Sample size for each study was calculated to detect the respective primary endpoint (either progression-free survival [PFS] or OS). It is important to note that these clinical trials were designed and completed before any validated stratification system for recurrence risk was established. Patient recruitment was mainly based on histopathological parameters and results may have been different if the SSIGN or UISS systems had been used for risk stratification.

Therapeutic vaccines also have been tested in adjuvant trials in RCC^23^ (see Table 1 for details). Autologous irradiated tumor cells mixed with bacillus Calmette-Guérin (BCG) was tested in two randomized trials and did not result in prolonged DFS.^24,25^ Similarly, autologous, tumor-derived heat-shock protein (glycoprotein 96)–peptide complex (HSPPC-96; vitespen) did not result in a statistically significant improvement of DFS.^26^ A trial with an autologous renal tumor cell vaccine only reported improved DFS in the vaccine group,^27^ but the number of patients lost after the randomization step, the imbalance of this loss, and the absence of tabulation of OS led to criticism of the results.^28^ This therapy has not been implemented in routine clinical practice.

In conclusion, immune therapy has no proven role in adjuvant therapy of RCC. Future autologous vaccine trials will need to be conservatively reported with the intention-to-treat analysis and the per-protocol analysis both reported to take into account the proportion of patients in whom an autologous vaccine cannot be manufactured.

### Completed Adjuvant Clinical Trials in RCC Using Hormone Therapy or Radiotherapy—The Past II

RCC has been considered potentially hormone-responsive since diethylstilbesterol was shown to induce malignant renal cancers in hamsters.^29^ Further animal studies demonstrated that RCC tumors were inhibited by hormone therapy. Additionally, 61% and 75% of RCCs have been reported to express estrogen and androgen receptors, respectively. However, treatment of RCC using hormone-directed therapy in patients has been largely unsuccessful with reported overall RRs between 2% and 6% in stage IV RCC.^29^ Despite these results, a prospective randomized multicenter study comparing adjuvant medroxyprogesterone acetate treatment for 1 year to no treatment following radical nephrectomy was conducted.^30^ Neither a significant difference in relapse rate nor correlations between receptors, relapses and treatment were found.

Radiotherapy can be useful for palliation of symptoms (eg, hematuria, painful bone metastases) in patients with stage IV RCC. Furthermore, long-term PFS has been reported for in a subset of patients following radiotherapy for solitary bone metastases.^31^ However, clinical trials evaluating the role of radiotherapy in the adjuvant setting revealed disappointing results. One prospective, randomized study in 72 patients comparing administration of 50 Gy in 20 fractions to the kidney bed, and ipsilateral and contralateral lymph nodes for stages II and III RCC versus observation reported relapse rates of 48% in both groups. Forty-four percent of patients in the radiotherapy arm had significant complications that contributed to the death of 19% of patients. The authors concluded that postoperative radiotherapy is without any beneficial effect on relapse rate and survival and is associated with an unacceptable complication rate.^32,33^

Consequently, neither hormone treatment nor radiotherapy is recommended as adjuvant therapy in patients with RCC. To our knowledge, neither treatment modality is currently being investigated in the adjuvant setting in clinical trials.

### Current Adjuvant Clinical Trials in RCC Using Targeted Therapies—The Present

While adjuvant clinical trials with immune-directed therapies did not demonstrate clinical benefit, they showed that large multicenter adjuvant clinical trials are feasible in RCC. Consequently, with the discovery of effective novel targeted therapies, a set of new well-powered adjuvant trials was designed (Table 2).

**Figure 2:**
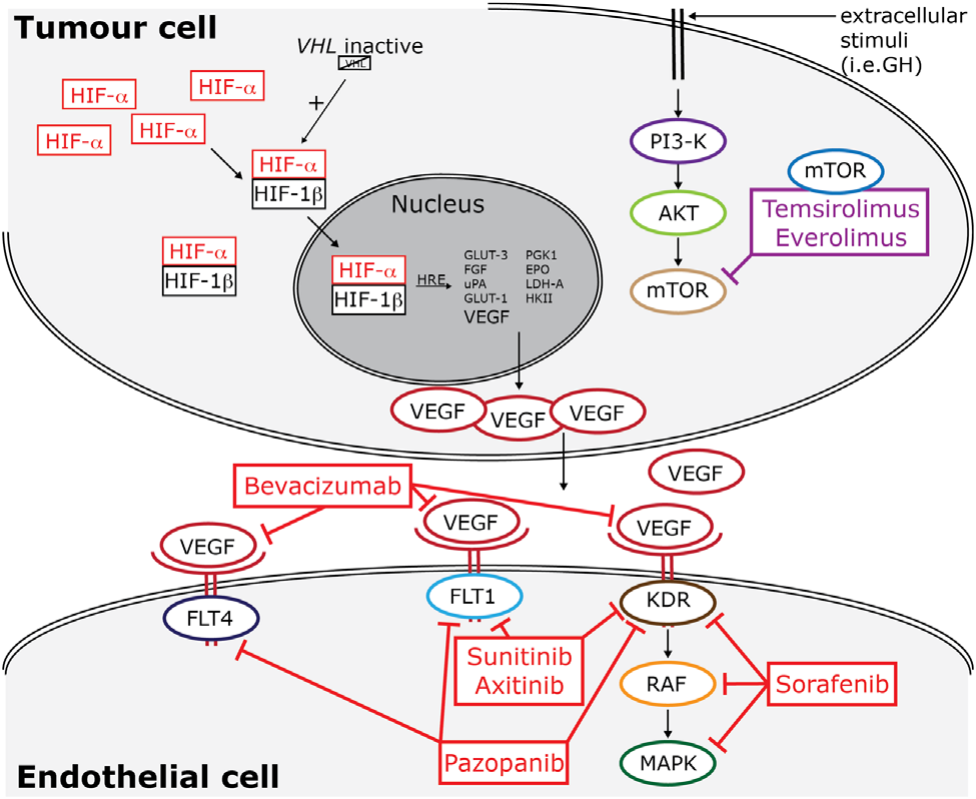
Important molecular pathways and drug targets in RCC. Inactive VHL leads to increased concentrations of HIF-alpha subunits. The PI3K/mTOR pathway also increases expression of HIF-alpha subunits. The resulting transcriptional stimulation of the HIF response element HRE and the rise in VEGF production stimulates endothelial growth and increased vascular supply to the tumor. Targeted therapies inhibit a variety of molecules along the involved signaling cascades in both tumor and endothelial cells. VHL,Von Hippel Lindau; HIF, hypoxia-induced factor; HRE, HIF response element; VEGF, vascular endothelial growth factor; mTOR, mammalian target of rapamycin; MAPK, mitogen-activated protein kinase. Adapted with permission of Future Medicine Ltd.^48^

The development of these targeted therapies has been informed by an improved understanding of the molecular biology and pathology of RCC, both in the tumor and in the surrounding epithelial cells (Figure 2). In the tumor cell, loss of the tumor-suppressor gene Von Hippel Lindau (*VHL*) (or its function),^34^ and the resulting downstream abnormalities, have been linked to clear cell RCC. VHL loss leads to increased activity of the hypoxia-induced factor (HIF) transcription factor^35^ and ultimately to overexpression of VEGF. HIF activity can also be increased via the mTOR pathway.^36^ Ultimately, this leads to increased tumor vascularity due to the endothelial response to VEGF. Rathmell and Nathanson have reviewed the molecular biology of RCC in more detail in this issue.

Various parts of these molecular pathways have been targeted successfully either by mTOR inhibitors such as temsirolimus and everolimus, or by bevacizumab, a monoclonal antibody to VEGF, or by intracellular inhibition of VEGF signaling in endothelial cells using TKIs (Figure 2). Multi-targeted TKIs, such as sorafenib, sunitinib, and pazopanib, target the intracellular kinase domains of VEGF receptors (VEGFR1–3), and, in the case of sorafenib, the downstream kinases RAF and MAPK.^37^

Clinical trials in stage IV RCC with VEGF TKIs and bevacizumab have shown significant improvements in PFS and OS and have led to the regulatory approval of these drugs to treat patients with stage IV RCC (Figure 1). They consequently form the main stem of clinical practice guidelines for first-line treatment in metastatic disease.^38^ Everolimus and axitinib have led to prolongation of PFS in second-line treatment after first-line treatment with VEGF antagonists^39,40^ (see Escudier and Gore in this issue of Seminars).

These small molecule targeted therapies (TKIs and mTOR inhibitors) have good oral bioavailability and therefore lend themselves particularly well to large multicenter adjuvant clinical trials. In the metastatic setting they most often lead to static disease and they are not cytotoxic to the tumor cell. This may reduce their efficacy in the adjuvant setting. Nevertheless, there are currently seven large, multicenter, placebo-controlled, double-blind, randomized adjuvant clinical trials are in progress in RCC. Five of them use TKIs, one an mTOR inhibitor, and one a monoclonal chimeric antibody (mAb) to carbonic anhydrase IX. These trials are listed alphabetically below and with more detail in Table 2.

#### ARISER (NCT00087022)

This trial investigates girentuximab, a mAb to carbonic anhydrase IX, a HIF downstream target gene. It has completed recruitment and has not met the primary endpoint. The treatment had no beneficial effect on DFS.^41^

#### ASSURE (NCT 00326898)

This trial has two experimental arms comparing sunitinib and sorafenib. The primary endpoint is DFS. The study is active but no longer recruiting and completion is estimated for 2016.

#### ATLAS (NCT01599754)

Patients in the experimental arm of this trial receive axitinib 5 mg twice daily. The primary endpoint is DFS. It started recruitment in April 2012 and is expected to complete for the primary end point of DFS in 2017. The study centers are in Japan and the study population is therefore East Asian. This may have an impact on the toxicity profile.

#### PROTECT (NCT01235962)

Patients in the experimental arm in this trial receive 800 mg of pazopanib daily. The primary endpoint is DFS after 4.5 years of follow-up and completion is expected for 2017.

#### SORCE (NCT00492258)

This trial has two experimental arms. Patients either receive sorafenib for 1 or 3 years. Relapse risk prior to trial enrollment is calculated using the SSIGN score. The primary endpoint is DFS. Enrollment is likely to be completed in 2013.

#### S-TRAC (NCT00375674)

Patients in the experimental arm of this trial receive sunitinib. The trial uses the UISS score for risk stratification. The primary endpoint is DFS and the primary analysis is expected in 2017.

#### SWOG-S0931 (EVEREST; NCT01120249)

This trial is the only large adjuvant trial investigating an mTOR inhibitor. Patients on the treatment arm of this trial receive 12 months of everolimus. The primary endpoint is DFS. The trial started in 2011 and is not expected to complete until 2021.

A number of smaller adjuvant clinical trials are also listed on the Clinical Trials register (www.clinicaltrials.gov), such as the AGuo trial (NCT01041482). This trial, for example, investigates sorafenib in a small cohort of patients using an open-label design. These trials may add to our understanding of adjuvant treatment in RCC, but they do not fulfill the requirements of high-level clinical evidence and therefore will not be discussed further in this publication.

As detailed in Table 2, a number of the six large adjuvant trials with small molecules have been open for several years. While official reports on endpoints are still outstanding, some important aspects have become apparent over time. In particular, trials with VEGF TKIs have had an unexpectedly high drop-out rate due to toxicity. The side effect profile of the TKIs used in these trials includes a number of off-target effects such as hypothyroidism, liver toxicity, palmar and plantar erythema, rash, mucositis, anorexia, tiredness, and diarrhea. These are observed both in the treatment of patients with stage IV disease and in the adjuvant setting but seem to be harder to tolerate in the adjuvant setting. They add to the on-target side effects of proteinuria and hypertension, mediated by endothelial remodelling. Most of the trial protocols exclude patients with uncontrolled hypertension or coronary artery disease in anticipation of endothelial side effects. The off-target effects and their severity, however, are more difficult to predict. In the SORCE trial, for example, an initial toxicity rate of 20% was predicted from sorafenib use in the advanced setting, but early analysis revealed a toxicity rate of 35%. ASSURE investigators have reported similar findings. Additionally, both trials have implemented a lead-in period of half-dose trial medication, as the majority of toxicities resulting in discontinuation of trial participation were observed in the first 12 weeks.

In this issue of *Seminars*, Kollmannsberger et al discuss the management of treatment-related toxicities and expert consensus also has been published by leading clinicians in the field in early 2012.^42^ More successful management of toxicity can be expected to have a positive impact on adjuvant clinical trial enrollment and drop-out rates. Equally, an understanding of the correct dosage for adjuvant treatment may help to reduce toxicity. Many of the current adjuvant trials use the same dose as licensed for palliative treatment of stage IV disease. Smaller doses may be sufficient to improve DFS and will reduce toxicity.

The potential long-term side effects of VEGF-targeted therapy remain unknown and will require prolonged follow-up from these trials. The potential for long-term unexpected side effects as seen in the adjuvant studies in breast cancer is apparent.

### Upcoming Adjuvant Trial Medications in RCC—The Future Part I

Translational efforts in adjuvant trials are based on the molecular preclinical and clinical understanding of the disease and on knowledge gained during development of the treatments in stage IV disease. The future adjuvant clinical trial landscape in RCC will therefore be informed by the increasing understanding of the molecular pathology of RCC and by the emerging clinical evidence for treatment of metastatic RCC.

A number of molecular drug targets have been identified and are in phase II/III clinical trials for stage IV RCC. In January 2013, ClinicalTrials.gov listed 139 open and active phase II/III clinical trials for RCC. A recent detailed review of these trials identified 11 novel targeted agents.^43^ These include new TKIs with in addition to Akt inhibitors. In preclinical studies, pathways such as the HIF pathway are of interest and may provide further drug targets.^44^ There is also evidence that novel immune therapy may contribute to the future treatments in RCC. Preliminary data suggest that an anti programmed death 1 protein ligand (PD-L1) antibody is safe and may induce tumor regression in RCC.^45^ Further immune therapies are discussed by McDermott and Atkins in this issue of *Seminars*.

Furthermore, there is an emerging interest in combination therapy in metastatic renal cancer (see Ravauld and Bellmunt this issue). This includes the combination of TKIs with immune therapies (eg, NCT01513187: Pazopanib with Interferon Alfa 2-A), combination of TKIs with chemotherapeutics (eg, NCT00556049: Sunitinib with Gemcitabine), and the combination of anti-VEGF antibodies and mTOR inhibitors (eg, NCT01399918: Bevacizumab and Everolimus). All of these treatments may be of interest for future adjuvant trials in RCC if they are found to be effective in stage IV disease. However, they may be more toxic, making them less suitable in particular for adjuvant treatment.

### Considerations Regarding Adjuvant Trial Designs in RCC—The Future Part II

The above list of new therapeutic approaches is not complete. However, even from these examples it is evident that there will be a multitude of potential candidate treatments and regimens for novel adjuvant clinical trials in RCC. As a consequence of the numerous possibilities for adjuvant clinical trials, challenges arise for adequate trial design and patient allocation to ensure sufficiently powered trials and interpretable results. In addition to the risk stratification systems discussed above, validated biomarkers of disease recurrence may facilitate patient allocation and stratification in future trials. These biomarkers could be cross-validated in adjuvant trials after initial implementation in interventional trials for stage IV disease. Both molecular markers and clinical markers of disease response are discussed in separate papers in this issue (see Garcia-Donas and Jonasch, and Michaelson and Stadler). Future adjuvant clinical trials should draw on knowledge of these markers and also should include translational endpoints to enable validation in the context of disease recurrence. Biomarkers and monitoring of drug levels also may guide prediction of toxicity profiles and could therefore contribute to reducing trial drop-out rates.

**Figure 3:**
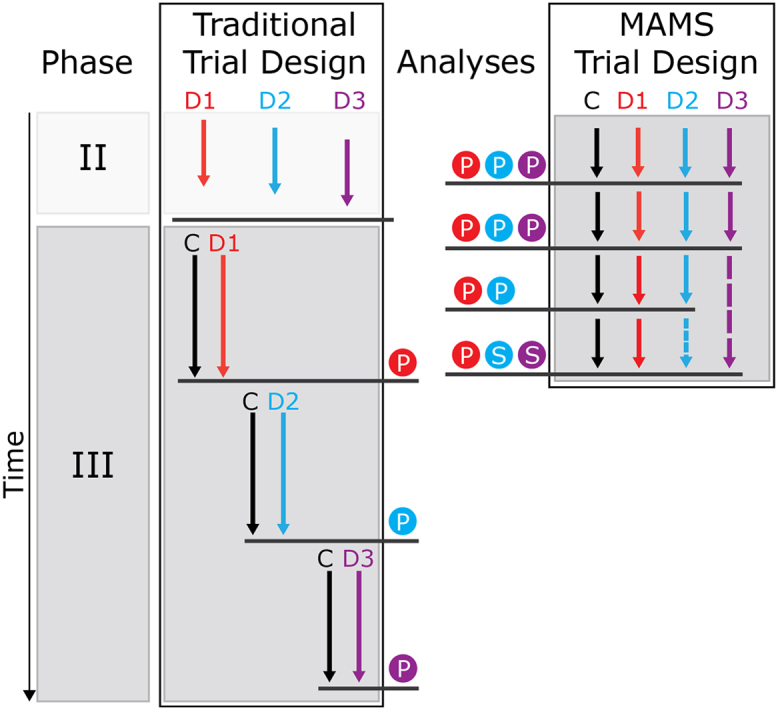
Schematic comparison of traditional and MAMS trial design. Testing three experimental treatments with traditional trial design requires more time and more control arm enrollment than MAMS trial design. Separate analysis and design of phase II and phase III trials makes the traditional trial design process also less efficient in terms of cost and administrative effort. Additional points of analysis allow for early termination of non-effective experimental treatments in MAMS trials. MAMS, multi-arm multi-stage; C, control/placebo arm; D, drug/experimental arm; P, point of primary analysis; S, point of secondary analyses. Adapted with permission of Future Medicine Ltd.^44^

The problem of multiple therapeutic hypotheses is, of course, not unique to RCC and future adjuvant clinical trials in RCC can be modeled on novel adjuvant and non-adjuvant trial designs in other diseases. Multi-arm multi-stage (MAMS) trial design, for example, offers an alternative to the traditional clinical trial design (Figure 3) of testing each therapy or combination therapy in separate trials.^46^ In a MAMS trial the experimental arms for several treatments share one control arm, therefore reducing the number of patients enrolled in nontreatment parts of clinical trials, which is also a significant advantage from the patient perspective. Early and repeated interim analyses that expedite discontinuation of ineffective treatments minimize the number of patients recruited to non-beneficial intervention. Importantly, early analysis allows timely discontinuation and publication of the trial, in case of sufficient evidence for efficacy. MAMS trial design can therefore deliver conclusive results on the multiple experimental treatments with significantly reduced enrolment numbers. In addition, MAMS trials reduce the time requirement for clinical trials by rolling phase II into phase III trials, providing the interim analysis is favorable. Biomarkers of relapse could shorten the required time period to interim analyses further. This also reduces the administrative effort and the cost for the clinical trial. Finance, ethics applications, and research and development applications all can be unified rather than being separately submitted for phase II and phase III.

These theoretical advantages are currently delivered in the Medical Research Council STAMPEDE trial, which compares hormone alone with a combination of hormone therapy and either zoledronic acid, docetaxel (or both), celecoxib, or abiraterone in prostate cancer.^47^ This trial had one treatment arm discontinued after interim analyses and also one addition of an experimental arm. It has thus demonstrated that MAMS trials are feasible and may accelerate progress if multiple treatment options are to be evaluated.

We suggest that future adjuvant clinical trials should incorporate the lessons from the past, such as anticipation of side effects, in addition to the innovation of new therapeutic discovery, novel biomarkers, and trial design. Considering the advances in these fields, it is very likely that the next few years will see growing interest in adjuvant clinical trials.

## CONCLUSION

Consensus guidelines recommend consideration of clinical trial enrollment to deliver potential adjuvant treatment to patients with intermediate or high relapse risk from early-stage RCC.^5^ These recommendations reflect the unmet requirement for relapse risk reduction. The need for improved adjuvant treatment is amplified by an increasing incidence of early stage RCC. Unfortunately, success in the treatment of metastatic RCC with immune therapy has not yet been successfully translated into adjuvant treatment. The results of seven large, multicenter, placebo-controlled, double-blind, randomized adjuvant trials investigating the potential benefit of targeted anti-angiogenesis therapies are expected over the coming years.

Further understanding of the molecular pathology of RCC and new therapeutic success in metastatic RCC will generate future hypotheses for adjuvant clinical studies. New immune therapies and successful combination therapies will add to this pool of suitable adjuvant candidate therapies. This potential wealth of therapies poses a novel challenge to the adjuvant trials setting. Multiple potential therapies will have to be assessed concurrently. Economy of time, cost, and most importantly patient enrollment will consequently be crucial to future trials. New strategies are therefore required, not only with regard to the biological and pharmacological approaches to relapse risk reduction in RCC but also with regard to trial design and resource allocation. Overall, the landscape of adjuvant clinical trials in RCC is likely to expand and diversify further in the years to come.

## Figures and Tables

**Table 1:** Adjuvant Trials in RCC Using Immune Therapies Ordered by Appearance in the Text

Authors	Intervention	Patient Population	Design	No. of Patients	Outcome
Pizzocarro et al^15^	IFN-α2b *v* placebo	Robson stages II and III (T3aN0M0 and T3bN0M0 or T2/3N1-3M0)	Multicenter, randomized, controlled trial	247	5-year OS: 0.665 (control) *v* 0.660 (treatment) (HR 1.040; 95% CI, 0.671 - 1.613, *P =* 0.861)
					5-year DFS: 0.671 (control) *v* 0.567 (treatment) (HR 1.412; 95% CI, 0.927–2.149, *P = .*107)
Messing et al^16^	IFN-α-NL *v* observation	pT3–4a and/or node-positive)	Multicenter, randomized, controlled trial	283	At 10.4 years median follow-up:
					Median survival: 7.4 years (control) *v* 5.1 years (treatment) (*P = .*09).
					DFS: 3.0 years (control) *v* 2.2years (treatment) (*P = .*33)
Clark et al^17^	IL-2 *v* observation	T3b-4 or N1–3 (LA) or M1	Multicenter, randomized, controlled trial	69 total; 44 LA, 25 M1 disease	2-year DFS: 48% (control in LA patients) *v* 53% (treatment in LA patients) (*P =* 0.73)
					2-year OS: 77% (control in LA patients) *v* 86% (treatment in LA patients) (*P = .*38)
Passalacqua et al^18^	IL-2 and IFN-α *v* observation	pT_1_, T_2_, T_3_ a-b-c; pN0-pN3, M0	Multicenter, randomized, controlled trial	310	5-year DFS: 0.73 (control) *v* 0.73 (treatment)
					10-year DFS: 0.60 (control) *v* 0.73 (treatment) (HR 0.84; 95% CI, 0.54–1.33, *P =* 0.47)
Atzpodien et al^19^	IL-2 and IFN-α2a and intravenous 5 *v* fluorouracil	pT3b/c pN0 or pT4pN0), pN, complete resection of tumor relapse or solitary metastasis (R0)	Multicenter, randomized, controlled trial	203	At median follow-up of 4.3 years:
					2-year OS: 91% (control) *v* 81% (treatment)
					5-year OS: 76% (control) *v* 58% (treatment)
					8-year OS: 66% (control) *v* 58% (treatment) (*P =* 0.0278)
					2-year DFS: 62% (control) *v* 54% (treatment)
					5-year DFS: 49% (control) *v* 42% (treatment)
					8-year DFS: 49% (control) *v* 39% (treatment) (*P =* 0.2398)
Aitchison et al^22^	IL-2 and IFN-α2a and intravenous 5-fluorouracil	T3b-c,T4 or any pT and pN 1 or pN 2 or positive microscopic margins or microscopic vascular invasion	Multicenter, randomized, controlled trial	309	3-year DFS: 50% (control) *v* 60% (treatment) (HR 0.87; 95% CI, 0.63-1.20)
					5 year OS: 60% (control) *v* 68% (treatment) (HR 0.91; 95% CI, 0.60–1.38)
Galligioni et al^24^	Autologous irradiated tumor cells & BCG *v* observation	Stages I, II, and III	Prospective, randomized, controlled trial	120	At 61 months median follow-up:
					5-year OS: 78% (control) *v* 69% (treatment)
					5-year DFS: 72% (control) *v* 63% (treatment)
Adler et al^25^	Autologous irradiated tumor cells & BCG & hormone *v* hormone	All stages	Prospective, randomized, controlled trial	43	Trend for prolongation of DFS for stage I, II, and III
					(*P*<.1)
Wood et al^26^	Autologous, tumor-derived heat-shock protein (glycoprotein 96)–peptide complex (HSPPC-96; vitespen) *v* observation	cT1b–T4 N0 M0, or cT any N1-2 M0	Multicenter, randomized, controlled trial	819	At 1.9 years median follow-up:
					Recurrence: 39.8% (control) *v* 37.7% (treatment) (HR 0·923; 95% CI, 0·729–1·169, *P =* .506)
					OS not mature
Jocham et al^27^	Autologous renal tumor cells (Reniale)	pT2–3b pN0–3 M0	Multicenter, randomized, controlled trial	558	At 5-year follow-up:
					DFS: 67.8% (control) *v*77.4% (treatment) (*P* = .0204) At 70-month follow-up:
					DFS: 59.3% (control) *v* 72% (treatment)
					HR for tumor progression: 1.58 (95% CI 1.05–2.37) and 1.59 (1.07–2.36) (*P = .*0204)

Note. The table includes trials with cytokines and vaccines. Abbreviations: IFN, interferon; IL, interleukin; NL, neutral lymphoblastoid; LA, locally advanced; BCG, bacillus Calmette-Guérin; CI, confidence interval; LA, locally advanced; HR, hazard ratio; M, metastatic; OS, overall survival; DFS, disease-free survival.

**Table 2:** Clinical Trials Database (http://clinicaltrials.gov) Listed Large, Multicenter, Placebo-Controlled, Randomized, Double-Blind Adjuvant Clinical Trials in RCC

**Acronym**	**Trial No.**	**Status**	**Intervention**	**Funding Body/Sponsors**	**Design**	**Start Date/ Estimated Completion**	**Stratification**	**Estimated Enrollment**	**Outcome Measures**

ARISER	NCT00087022	Reported negative, press release only	Girentuximab	Industry; Wilex	MC, DB, R, PC	07/2004	High-risk patients based on TN stage or Fuhrman grade, ECOG PS= 0 or 1	864	Primary endpoint, DFS, not met
						10/2012			
ASSURE	NCT00326898	Active, not recruiting	Sorafenib or Sunitinib	NIH; NCI, ECOG, SWOG, Cancer and Leukemia Group B, NCIC	MC, DB, R, PC	05/2006	At least intermediate high-risk UISS, ECOG PS= 0 or 1, clear or non-clear cell RCC	1923	DFS, OS, Toxicity, QoL
						04/2016			
ATLAS	NCT01599754	Recruiting	Axitinib	Industry; SF J Pharmaceuticals, Pfizer	MC, DB, R, PC	04/2012	High-risk UISS, ECOG PS= 0 or 1, predominant clear cell histology	592	DFS, OS, Toxicity
						05/2019			
PROTECT	NCT01235962	Recruiting	Pazopanib	Industry; GlaxoSmithKline	MC, DB, R, PC	11/2010	Modified UISS, Karnofsky performance scale of at least 80, clear cell or predominant clear cell histology	1500	OS, DFS, Toxicity, QoL
						04/2017			
SORCE	NCT00492258	Recruiting	Sorafenib	Medical Research Council UK	MC, DB, R, PC	05/2007	Intermediate- and high-risk SSIGN, ECOG PS= 0 or 1, clear or non-clear cell RCC	1656	OS, DFS, Toxicity
						2013			
S-TRAC	NCT00375674	Active, not recruiting	Sunitinib	Industry; Pfizer	MC, DB, R, PC	07/2007	High risk UISS, ECOG PS= 0-2, predominant clear cell histology	720	DFS, OS, Toxicity
						06/2017			
SWOG-S0931	NCT01120249	Recruiting	Everolimus	NIH; SWOG, NCI	MC, DB, R, PC	04/2011	Pathological high or very high risk, no further details available, ECOG PS= 0 or 1	1218	OS, DFS, Toxicity

Abbreviations: ARISER, Adjuvant Rencarex Immunotherapy phase III trial to Study Efficacy in non-metastatic RCC; ASSURE, Adjuvant Sorafenib or Sunitinib for Unfavorable Renal Carcinoma; ATLAS, Adjuvant Axitinib Treatment of Renal Cancer: a Randomized Double-Blind Phase 3 Study of Adjuvant Axitinib *v* Placebo in Subjects at High Risk of Recurrent RCC; PROTECT, a Randomized, Double-Blind, Placebo-Controlled Phase III Study to Evaluate the Efficacy and Safety of Pazopanib as Adjuvant Therapy for Subjects With Localized or Locally Advanced RCC Following Nephrectomy; SORCE, a Phase III Randomized Double-Blind Study Comparing Sorafenib With Placebo in Patients With Resected Primary Renal Cell Carcinoma at High or Intermediate Risk of Relapse; S-TRAC, Sunitinib Treatment of Renal Adjuvant Cancer: a Randomized Double Blind Phase 3 Study of Adjuvant Sunitinib *v* Placebo in Subjects at High Risk of Recurrent RCC; SWOG-S0931, EVEREST, EVErolimus for Renal Cancer Ensuing Surgical Therapy, a Phase III Study; DB, double blind; ECOG, Eastern Cooperative Oncology Group; DFS, disease-free survival; PS, performance score; MC, multicenter; NCI, National Cancer Institute; NIH, National Institute of Health; OL, open label; OS, overall survival; PC, placebo-controlled; QoL, quality of life; SWOG, Southwest Oncology Group; R, randomized; TN, tumor, node; UISS, UCLA Integrated Staging System.
